# Skeletal muscle–specific eukaryotic translation initiation factor 2α phosphorylation controls amino acid metabolism and fibroblast growth factor 21–mediated non–cell-autonomous energy metabolism

**DOI:** 10.1096/fj.15-275990

**Published:** 2015-10-20

**Authors:** Masato Miyake, Akitoshi Nomura, Atsushi Ogura, Kenji Takehana, Yoshihiro Kitahara, Kazuna Takahara, Kazue Tsugawa, Chinobu Miyamoto, Naoko Miura, Ryosuke Sato, Kiyoe Kurahashi, Heather P. Harding, Miho Oyadomari, David Ron, Seiichi Oyadomari

**Affiliations:** *Division of Molecular Biology, Institute for Genome Research, and ^†^Department of Molecular Research, Diabetes Therapeutics and Research Center, The University of Tokushima, Tokushima, Japan; ^‡^Exploratory Research Laboratories, Research Center, Ajinomoto Pharmaceuticals Company, Limited, Kanagawa, Japan; and ^§^Cambridge Institute for Medical Research, University of Cambridge, Cambridge, United Kingdom

**Keywords:** ER stress, ATF4, glutathione, obesity, small molecule

## Abstract

The eukaryotic translation initiation factor 2α (eIF2α) phosphorylation-dependent integrated stress response (ISR), a component of the unfolded protein response, has long been known to regulate intermediary metabolism, but the details are poorly worked out. We report that profiling of mRNAs of transgenic mice harboring a ligand-activated skeletal muscle–specific derivative of the eIF2α protein kinase R-like ER kinase revealed the expected up-regulation of genes involved in amino acid biosynthesis and transport but also uncovered the induced expression and secretion of a myokine, fibroblast growth factor 21 (FGF21), that stimulates energy consumption and prevents obesity. The link between the ISR and FGF21 expression was further reinforced by the identification of a small-molecule ISR activator that promoted *Fgf21* expression in cell-based screens and by implication of the ISR-inducible activating transcription factor 4 in the process. Our findings establish that eIF2α phosphorylation regulates not only cell-autonomous proteostasis and amino acid metabolism, but also affects non–cell-autonomous metabolic regulation by induced expression of a potent myokine.—Miyake, M., Nomura, A., Ogura, A., Takehana, K., Kitahara, Y., Takahara, K., Tsugawa, K., Miyamoto, C., Miura, N., Sato, R., Kurahashi, K., Harding, H. P., Oyadomari, M., Ron, D., Oyadomari, S. Skeletal muscle–specific eukaryotic translation initiation factor 2α phosphorylation controls amino acid metabolism and fibroblast growth factor 21–mediated non–cell-autonomous energy metabolism.

Disruption of endoplasmic reticulum (ER) function impedes the folding of proteins synthesized in the ER and leads to ER stress. During ER stress, the unfolded protein response (UPR) maintains ER proteostasis through translational and transcriptional regulation (1, 2). The UPR signal generated by ER stress is transmitted to the cytosol and nucleus through independent signaling pathways mediated by the 3 ER membrane proteins: inositol-requiring enzyme (IRE)1, protein kinase R-like ER kinase (PERK), and activating transcription factor (ATF)6. Studies conducted over the past decade demonstrate that UPR signaling communicates with various metabolic signaling pathways, including those triggered by insulin signaling (3, 4). Thus, ER stress is now recognized as a contributor to metabolic disorders such as obesity and diabetes mellitus.

PERK phosphorylates Ser^51^ of the α subunit of eukaryotic translation initiation factor 2 (eIF2α), resulting in a reduction in the rates of translation initiation. This phosphorylation event also induces gene expression for several transcription factors, including ATF4 (5–7). Mammalian cells have 4 different eIF2α kinases that recognize a different set of stress conditions. For example, PERK is activated, not only by ER stress, but also by various other stressors, such as general control nonderepressible (GCN)2 activation caused by amino acid deficiency, heme-regulated inhibitor kinase activation caused by heme deficiency, and protein kinase R (PKR) activation caused by viral infection. Given its role in the stress response, the eIF2α signaling pathway is called the integrated stress response (ISR) (8, 9).

Skeletal muscles play an important role in metabolic homeostasis, as they are a major site of glucose utilization and for the storage of nutrients, such as amino acids and glycogen. In skeletal muscles, it has been shown that exercise activates the 3 ER stress-response pathways (10) and that aging activates the IRE1 pathway (11), suggesting that ER stress occurs in skeletal muscles under physiologic conditions. Muscle differentiation is initiated with the fusion of myoblasts to form multinucleated myotubes, which group together to form muscle fibers and then mature into contractile myofibers. The X-box binding protein (XBP)1 and ATF4 transcription factors act downstream of the IRE1 and PERK pathways, respectively, and negatively regulate muscle differentiation. XBP1 inhibits myotube formation (12), and ATF4 induces muscle fiber atrophy (13). Conversely, the ATF6 pathway promotes muscle differentiation by inducing apoptosis in immature myoblasts (14). Thus, given that the UPR pathways regulate completely opposing biologic functions, it is very important to clearly define the roles of each pathway.

Available evidence has led researchers to speculate that ER stress induced by certain factors in skeletal muscles activates UPR, which globally regulates skeletal muscle function and metabolism. However, because all 3 UPR pathways are simultaneously activated during ER stress, it is difficult to determine the influence of each pathway individually. Fv2E-PERK, which is a fusion protein of artificial dimerization domain (Fv2E) and PERK kinase domain, enables ligand-dependent PERK activation that is uncoupled from ER stress. In mice expressing an Fv2E-PERK transgene that specifically induces organ-specific and dose-dependent activation of the PERK pathway, we found that activation of the PERK pathway in the liver regulates carbohydrate and lipid metabolism (15). In the present study, we discovered 2 effects of skeletal muscle–specific activation of the PERK pathway by using the Fv2E-PERK system: PERK activation affects the level of amino acids necessary for antioxidant synthesis, and PERK activation confers some resistance to diet-induced obesity through the secretion of fibroblast growth factor (FGF)21 as a myokine that promotes energy consumption in brown adipose tissue (BAT). Furthermore, we identified a new small molecule by using cell-based screening that phosphorylates eIF2α and regulates downstream target genes related to amino acid metabolism as well as *Fgf21*. Collectively, our findings suggest that eIF2α phosphorylation in skeletal muscle cells control cell autonomous and non–cell-autonomous metabolic regulation, providing potential therapeutic targets for metabolic diseases.

## MATERIALS AND METHODS

### Transgenic mice

A human skeletal muscle α-actin promoter DNA fragment (2.2 kb; a gift from E. Hardeman, University of New South Wales, Sydney, NSW, Australia) was used to develop transgenic (TG) mice that specifically express Fv2E-PERK in skeletal muscles (16). Two TG lines, TG and TG (high) were established in the C57/BL6 strain. The mice were fed a standard diet containing 12% fat or a high-fat diet (HFD) containing 60% fat (Research Diets, Brunswick, NJ, USA) and were raised in specific pathogen-free conditions at the Institute of Genome Research of The University of Tokushima. The mice were given AP21087 (AP; Ariad Pharmaceuticals, Cambridge, MA, USA) or vehicle (4% ethanol, 10% polyethylene glycol-400, and 1.75% Tween-20 in water) by intraperitoneal injection. The Animal Research Committee of the University of Tokushima approved the study.

### Physiologic analysis

Body fat composition was measured with a LaTheta LCT-200 animal computed tomography (CT) scanner (Aloka, Tokyo, Japan). Energy expenditure was measured by indirect calorimetry over 2 consecutive days with the Comprehensive Lab Animal Monitoring System (Columbus Instruments, Columbus, OH, USA). Food intake was measured every 24 h. Grip strength was measured with an MK-380CM/R grip-force meter (Muromachi Kikai, Tokyo, Japan). The endurance of mice trained to run on a TMS-4 treadmill (Muromachi Kikai) was evaluated by determining how long and far they ran until they could not continue despite mechanical prodding. Two days before the endurance test, mice that had acclimated to running on the treadmill were tested in a treadmill program that increased the speed by 1 m/min every 10 min from the start to 30 min and then by 1 m/min every 5 min thereafter. For cold exposure, mice were placed at 4°C for 8 or 24 h. For the glucose tolerance test, mice were not fed overnight (14 h) and were injected with 2 g/kg i.p. glucose solution. For the insulin tolerance test, mice were not fed for 4 h and were injected with 0.75 mU/kg i.p. human insulin . Blood glucose levels were measured in tail blood with a OneTouch Ultra glucometer (LifeScan, Milpitas, CA, USA).

### Histologic analysis

Paraffin-embedded sections were prepared from mouse liver, fixed in 4% paraformaldehyde, and stained with hematoxylin and eosin (H&E). Frozen sections that were prepared from the liver were fixed in 4% paraformaldehyde, embedded in optimal cutting temperature compound, and stained with Oil Red O. Sections prepared from skeletal muscles frozen in dry-ice acetone were immunostained with anti-MyHC-slow (Sigma-Aldrich, Tokyo, Japan).

### Biochemical analyses

The plasma and liver triglycerides, plasma cholesterol, and plasma FGF21 concentrations were measured with the Triglyceride E-Test and Cholesterol E-Test (both from Wako Pure Chemicals, Osaka, Japan) and Mouse/Rat FGF-21 Quantikine ELISA kit (R&D Systems, Minneapolis, MN, USA), respectively. The intracellular amino acid concentration was measured with the LaChrom Elite HPLC System (Hitachi High Technologies, Tokyo, Japan). This system isolates amino acids from extracts prepared from cells treated with stable isotopes [ring-D5]-phenylalanine being used for normalization. The MSQ Plus LC/MS System (Thermo Fischer Scientific, Waltham, MA, USA) was used to identify the amino acids. Glutathione (GSH) concentrations in skeletal muscle homogenates (prepared with 5% 5-sulfosalicylic acid dehydrate) were measured with the oxidized glutathione (GSSG)/GSH Quantification kit (Dojindo Laboratories, Kumamoto, Japan). Mitochondrial DNA copy number was measured with the Step One Real-Time PCR System (Thermo Fisher-Applied Biosystems, Foster City, CA, USA). Total DNA, which was used as a template, was extracted from skeletal muscles *via* the standard proteinase K method.

### Cell culture

C2C12 myoblasts were cultured in DMEM with 10% fetal bovine serum and then for 3 d in DMEM with 2% horse serum in a collagen-coated dish, to induce muscle differentiation. Luciferase reporter experiments or other experiments were conducted 1 or 3 d after differentiation. *Atf4*^−/−^ mouse embryonic fibroblasts (MEFs) were cultured in DMEM with nonessential amino acids and 55 mM 2-ME. Plasmids were introduced into the cells by transfection with polyethylenimine (Polysciences, Warrington, PA, USA), by electrotransfection with Neon (Thermo Fisher–Life Technologies), and by transduction with the pCDF1 Lentivirus System (System Biosciences, Mountain View, CA, USA). Cells transfected with the Fv2E-PERK expression vector were selected by using hygromycin B or puromycin to obtain cells that stably expressed Fv2E-PERK. ER stress was induced by adding 2 μg/ml tunicamycin (Wako Pure Chemicals), 0.2 μM thapsigargin (EMS-Millipore, Billerica, MA, USA), 10 μg/ml brefeldin A (Wako Pure Chemicals), 1 mM DTT (Wako Pure Chemicals), or 0.5 μM integrated stress response inhibitor (ISRIB) to the culture medium.

### Metabolic flux assay of intact cells

C2C12 cell mitochondrial respiration, basal respiratory capacity, coupling efficiency, proton leakage, and spare respiratory capacity were profiled by an XF24 metabolic flux analyzer (Seahorse Bioscience, Billerica, MA, USA). Furthermore, the oxygen consumption rate (OCR) was assessed in C2C12 cells that had been induced to undergo differentiation while being continuously treated with the ATP synthase inhibitor oligomycin (Wako Pure Chemicals), the uncoupling agent carbonyl cyanide *p*-(trifluoromethoxyl)-phenyl-hydrozone (FCCP; StressMarq Biosciences Inc., Victoria, BC, Canada), and a mitochondrial complex inhibitor rotenone (Sigma-Aldrich)/antimycin (Enzo Life Sciences, Plymouth Meeting, PA, USA). Fatty acid β-oxidation was evaluated by measuring the OCR when differentiated C2C12 cells were treated with bovine serum albumin–conjugated palmitate after 1 h of equilibration in Krebs-Henseleit buffer containing 0.5 mM carnitine (Tokyo Chemical Industry, Tokyo, Japan) and 2.5 mM glucose.

### Luciferase promoter assay

C2C12 cells were transfected with the luciferase reporter vectors containing the murine *Fgf21* promoter and vectors expressing effectors for either Fv2E-PERK or ATF4. These cells were then induced to differentiate as described above, and luciferase activity was measured with the One-Glo Luciferase Assay System (Promega, Madison, WI, USA).

### RNA analysis

Total RNA from cells and tissue samples were used as templates for cDNA synthesis with ReverTra Ace qPCR RT Master Mix with gDNA Remover (Toyobo, Osaka, Japan). The Prism 7900HT Real-Time PCR System (Thermo Fisher–Life Technologies), Step One Plus Real-Time PCR System (Thermo Fisher–Life Technologies) with Thunderbird qPCR Mix (Toyobo) or FastStart Universal SYBR Green Master (Roche Diagnostics, Tokyo, Japan) were used for quantitative PCR (qPCR) analyses. *Actb* (β-actin) for skeletal muscles, *Rplp0* (36B4) for BAT, and *Gapdh* (glyceraldehyde phosphate dehydrogenase) for cultured cells served as internal controls. Before performing microarray analysis, the Low Input Quick Amp Labeling Kit (Agilent Technologies, Santa Clara, CA, USA) was used to label total RNA purified with the RNeasy MinElute Cleanup Kit (Qiagen, Tokyo, Japan). The labeled RNA was then used to probe a SurePrint G3 Mouse Gene Expression 8 × 60K Microarray, and the signals were scanned with a G2565 microarray scanner (both from Agilent Technologies). Microarray data were extracted from the scanned image with Feature Extraction 10.7 (Agilent Technologies), and the raw unfiltered microarray data were deposited in the Gene Expression Omnibus dataset (subseries entries GSE46548; *http://www.ncbi.nlm.nih.gov/geo/*). The isolation and analysis of differentially expressed genes was performed on the Subio Platform (Subio, Tokyo, Japan). GO enrichment and promoter analyses were performed with the Genomatix Genome Analyzer (Genomatix, Tokyo, Japan).

### Protein analysis

Total cell lysates and cytoplasmic and nuclear extracts were treated with protease inhibitor cocktail (Nacalai Tesque, Kyoto, Japan) and PhosStop Phosphatase Inhibitor Cocktail (Roche Diagnostics). Protein concentration was measured by the bicinchoninic acid method, and immunoblot analysis was performed (17). Proteins were detected with anti-PERK, anti-eIF2α, and anti-ATF4 (Santa Cruz Biotechnology, Dallas, TX, USA); anti–phospho-eIF2α, anti-AMPK, anti–phospho-AMPK, anti–acetyl-CoA carboxylase (ACC), anti–phospho-ACC, anti–eIF4E-binding protein (4EBP)1, and anti–phospho-4EBP1 (Cell Signaling Technology, Beverly, MA, USA), and anti-GRP78 (BD Bioscience, Tokyo, Japan) antibodies. The bands were detected with Immobilon Western Chemiluminescent HRP Substrate (EMS-Millipore), and the images were acquired with the EZ-Capture II Cooled CCD Camera System (ATTO Corp., Tokyo, Japan).

### Chromatin immunoprecipitation

Cells were fixed with 1% formaldehyde for 10 min. After the addition of 1.5 M glycine, the cells were lysed with SDS-lysis buffer [50 mM Tris-HCl (pH 8.0), 1% SDS, 10 mM EDTA with protease inhibitor cocktail] and sonicated with a Bioruptor sonicator (Cosmo Bio, Tokyo, Japan) for five 30 s pulses. Sonicated samples were diluted with dilution buffer [50 mM Tris-HCl (pH 8.0), 167 mM NaCl, 1.1% Triton X-100, and 0.11% sodium deoxycholate with a protease inhibitor cocktail] and preincubated with 50% protein G Sepharose for 2–6 h. After centrifugation, supernatants were incubated with 2.5 μg of ATF4 antibodies (Santa Cruz Biotechnology) or control rabbit IgG overnight at 4°C and with 50% protein G Sepharose for 2–3 h. The immunoprecipitates were sequentially washed once each with a low-salt buffer, a high-salt buffer, and an LiCl buffer and twice with a Tris-EDTA buffer. The DNA-protein complex was eluted by heating at 65°C for 6 h. RNA was removed by the addition of RNase A, and proteins were digested with proteinase K. DNA was collected by phenol-chloroform methods. Quantification was performed with FastStart Universal SYBR Green Master (Roche Diagnostics).

### Nonradioactive measurements of protein synthesis

To measure protein synthesis in skeletal muscle *in vivo*, we used the nonradioactive surface sensing of translation (SUNsET) technique (18). In brief, mice were injected with 0.04 μmol/g i.p. puromycin dissolved in PBS. At 30 min after injection, the gastrocnemius muscles were excised and frozen in liquid N_2_. Total protein was extracted from samples, and immunoblot analysis was performed with an anti-puromycin antibody (Kerafast, Boston, MA, USA).

### Chemical screening

Human embryonic kidney (HEK)293A cells were transfected with a pGL4-3xAARE luciferase reporter vector containing the hygromycin-resistance gene. The cells were selected with hygromycin after limiting-dilution cloning. One clone was used in this study. The cells were seeded onto 96-well plates and treated with each chemical and beetle luciferin (Promega) for 16 h. Luciferase activity was measured with EnVision (Perkin Elmer, Waltham, MA, USA). Secondary screening in C2C12 cells was performed by the same methods as were used for the *Fgf21* promoter assay.

### Plasmid construction

*Fv2E-Perk* and *Atf4* were cloned into the pCDF1-MCS2-EF1-Puro lentiviral expression vector (System Biosciences), pEBMulti-Hyg (Wako Pure Chemicals), and pcDNA3.1 (Thermo Fisher–Life Technologies). Regions upstream of the murine *Fgf21* transcription initiation site (−1326 to +100, −950 to +100, and −110 to +100) were cloned into pGL3 luciferase reporter vectors (Promega). PCR-based site-directed mutagenesis was used to generate AARE1 and -2 mutants. The DNA sequence of each construct was verified on an ABI 3130 DNA sequencer (Thermo Fisher–Applied Biosystems).

### Statistical analysis

All results are expressed as means ± sem. Unpaired 2-tailed Student’s *t* tests were performed to determine probabilities for paired samples, and 2-way ANOVA with repeat measurements was performed to analyze the kinetics data.

## RESULTS

### Physiologic activation of UPR pathways in skeletal muscles

**Figure 1.**
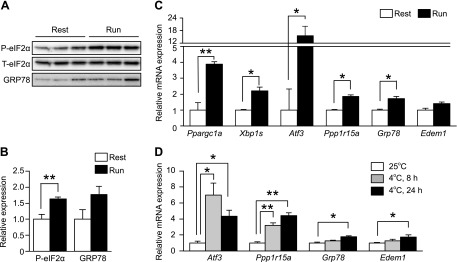
eIF2α is phosphorylated in mouse skeletal muscles under physiologic conditions*. A*) Representative immunoblots of phosphorylated and total eIF2α and GRP78 in the lower-limb skeletal muscles of mice during rest and at 4 h after 1 session of running on a treadmill*. B*) Densitometry quantification of phosphorylated eIF2α and GRP78 normalized to total eIF2 (*n* = 3–4). *C*) RT-qPCR analysis (*n* = 5) of *Ppargc1a* and the genes related to the UPR in the lower-limb skeletal muscles of mice at rest and at 5 h after 1 session of running on a treadmill*. D*) RT-qPCR analysis (*n* = 5) of the genes related to the UPR in the gastrocnemius muscles of WT mice exposed to 4°C for the indicated time. Means ± sem. *P < 0.05, **P < 0.01 *vs.* untreated mice.

Physiologic activation of UPR pathways has been reported in skeletal muscles by several groups (10, 19). To evaluate the occurrence of ER stress in skeletal muscles, we monitored the UPR downstream target genes during exercise and cold exposure in mice. When the mice were exercised for 4 h by treadmill running, the expression of peroxisome proliferative-activated receptor, gamma, coactivator 1 α (*Ppargc1a*), *Atf3*, protein phosphatase 1 regulatory subunit 15a (*Ppp1r15a*), glucose-regulated protein 78 (*Grp78*), and ER degradation enhancer, mannosidase α-like 1 (*Edem1*) was induced. In addition, slicing of *Xbp1* mRNA, up-regulation of eIF2α phosphorylation, and induction of GRP78 were observed. These findings are consistent with the results of previous reports (**Fig. 1*A*–*C***). When the mice were maintained at 4°C, the mRNA expression of *Atf3*, *Ppp1r15a*, *Grp78,* and *Edem1* was induced. Among the UPR downstream target genes, PERK pathway targets (*Atf3* and *Ppp1r15a)* showed more significant induction than the IRE1 and ATF6 pathway targets (*Grp78*, and *Edem1*) (Fig. 1*D*). Thus, we confirmed that, in skeletal muscles, ER stress occurs under physiologic conditions.

### Generation and phenotyping of skeletal muscle–specific activation of the PERK pathway

**Figure 2.**
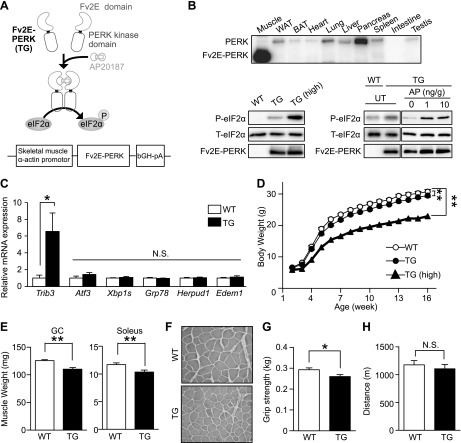
Constitutive phosphorylation of eIF2α in mouse skeletal muscles decreases body weight*. A*) AP-dependent eIF2α phosphorylation by Fv2E-PERK. Fv2E-PERK comprises an Fv2E domain that undergoes AP-dependent dimerization and a PERK domain that is activated upon dimerization. Fv2E-PERK phosphorylates eIF2α in response to AP treatment. *B*) Immunoblots of phosphorylated eIF2α, total eIF2α, and Fv2E-PERK in WT, TG and TG (high) mice, respectively. Top: tissue distribution of Fv2E-PERK in TG mice. Bottom left: immunoblots of Fv2E-PERK and phosphorylated and total eIF2α in gastrocnemius muscles of TG and TG (high) mice. Bottom: immunoblots of Fv2E-PERK and phosphorylated, and total eIF2α in gastrocnemius muscles of WT and TG mice treated with AP. UT means untreated*. C*) RT-qPCR analysis (*n* = 5) of the genes targeted by each UPR branch in gastrocnemius muscles of WT and TG mice*. D*) Body weights (*n* = 10–13; 2-way ANOVA) of WT, TG, and TG (high) mice fed a standard diet*. E*) Muscle weight (*n* = 12–13) of the gastrocnemius and soleus muscles of WT and TG mice fed a standard diet. *F*) Representative H&E staining of the tibialis anterior of WT and TG mice fed a standard diet. Grip strength (*n* = 13) (*G*) and treadmill endurance (*n* = 4–7) (*H*) of WT and TG mice fed a standard diet. Means ± sem. *P < 0.05, **P < 0.0 *vs.* WT mice.

Three UPR pathways were activated in physiologic stress conditions, as described above. To assess the significance of the activation of a single PERK pathway without the effect of the other 2 pathways, we used a skeletal muscle α-actin promoter to generate transgenic (TG) mice that expressed Fv2E-PERK exclusively in muscle (**Fig. 2*A***). Fv2E-PERK had been used to activate PERK-eIF2α signaling upon the addition of the synthetic dimerizing molecule AP. Two lines of transgenic mice, which differentially expressed Fv2E-PERK, were generated. In this study, the low-expressing line was called TG mice and the high-expressing line was called TG (high). Fv2E-PERK was expressed only in the skeletal muscles of TG mice (Fig. 2*B*). When these TG mice were given the ligand AP, the PERK substrate eIF2α was phosphorylated in proportion to the dose of the ligand (Fig. 2*B*). Because the skeletal muscle α-actin promoter highly overexpressed Fv2E-PERK compared with endogenous PERK, the up-regulation of eIF2α phosphorylation was observed in these mice compared with the wild-type (WT) mice, even in the absence of AP. Although eIF2α phosphorylation was found to induce translational attenuation, protein synthesis in both Fv2E-PERK TG and TG (high) mice was comparable to that in WT mice (Supplemental Fig. 1*A*). Constitutive enhancement of eIF2α phosphorylation was found to induce one of the PERK pathway target genes (*Trib3*) but not IRE1 and ATF6 pathway target genes (*Xbp1*, *Grp78*, *Herpud*, and *Edem1*) in Fv2E-PERK TG mice, suggesting that the levels of eIF2α phosphorylation in the mice were within the range of physiologic stress conditions in muscle (Fig. 2*C*). Therefore, unless otherwise specified, our analyses of TG mice were performed without the administration of AP.

To determine the effect of constitutive PERK activation in mouse skeletal muscles, we monitored the growth rate among mice with different Fv2E-PERK expression levels. Body weight was slightly lower in TG mice and much lower in TG (high) mice than in WT mice (Fig. 2*D*). Apart from the slight gene-dosage–dependent decrease in body weight, TG mice were almost comparable to their WT littermates when fed a normal diet. Next, we sought to determine whether constitutive PERK activation affects the quantity and quality of skeletal muscles. TG mice had consistently smaller gastrocnemius and soleus muscle fibers (Fig. 2*E*, *F*). Accordingly, the grip strength of TG mice was weaker than that of WT mice (Fig. 2*G*). TG (high) mice showed greater muscle atrophy and weaker grip strength than TG mice, indicating gene dosage dependence (Supplemental Fig. 1*B*–*D*). However, the endurance capacity of TG mice was equivalent to that of WT mice, suggesting that constitutive PERK activation might protect skeletal muscles from exercise-induced damage (Fig. 2*H*).

### Phosphorylation of eIF2α in skeletal muscles promotes an increase in the levels of free amino acids and the antioxidant glutathione

**Figure 3.**
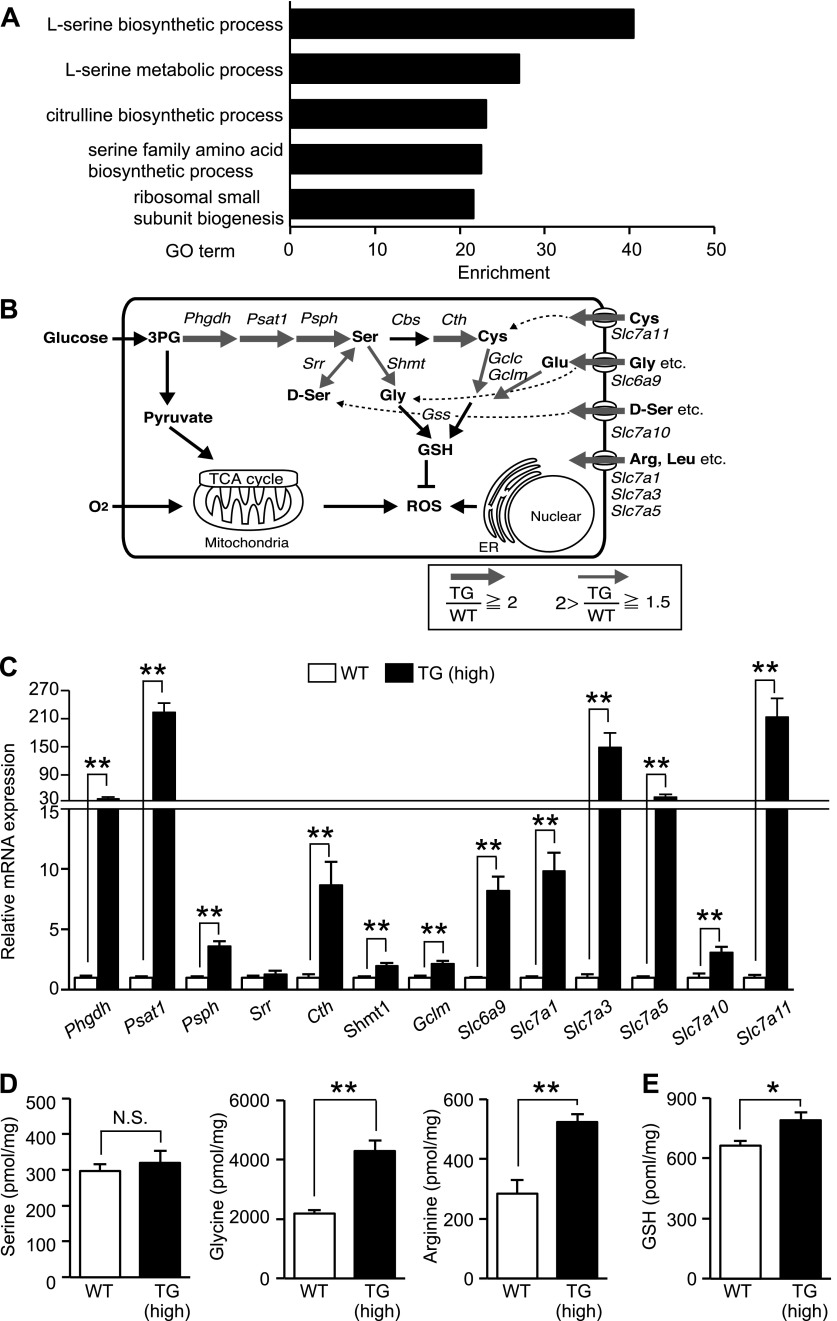
Phosphorylation of eIF2α increased GSH through transcriptional induction of amino acid metabolic genes in skeletal muscles*. A*) Analysis of gene ontology enrichment related to amino acid metabolism of elevated gene expression in skeletal muscles of TG mice*. B*) Changes in the expression of genes involved in amino acid synthesis and genes encoding amino acid transporters involved in GSH synthesis. The expression of these genes was detected in the skeletal muscle of TG mice in the presence of elevated levels of phosphorylated eIF2α and as induced gene clusters in C2C12 cells identified by microarray analysis. Red arrows: the expression of genes induced by eIF2α phosphorylation (thickness of the arrows is proportional to the degree of induction)*. C*) RT-qPCR analysis (*n* = 6) of the expression of mRNAs encoding proteins involved in amino acid metabolism that were detected in the gastrocnemius muscles of WT and TG (high) mice (8–10 wk old) fed a standard diet. Quantitation of free amino acid concentrations (*n* = 6) (*D*) and total GSH levels (*n* = 3–4) (*E*) in the gastrocnemius muscles of WT and TG (high) mice (8–10 wk old) fed a standard diet. Means ± sem. *P < 0.05, **P < 0.01 *vs*. WT.

Activated PERK phosphorylates eIF2α, which is known to lead to translational attenuation and transcriptional induction. We speculated that eIF2α phosphorylation takes part in the gene expression program that is necessary for stress adaptation in skeletal muscles. We therefore compared the genome-wide gene expression profile of TG (high) skeletal muscles to that of WT skeletal muscles. Gene ontology analysis revealed that genes related to amino acid metabolism were enhanced after eIF2α phosphorylation, with those involved in serine metabolism being particularly enriched in TG (high) mice compared with those in WT mice (**Fig. 3*A*, *B***). The previous studies in MEFs revealed the induction of an arginine and leucine transporter, solute carrier family 7A (*Slc7a*)*5*, and the glycine transporter *Slc6a9* (5). In this study, induction of a larger subset of genes involved in amino acid transport and metabolism was confirmed by RT-qPCR (Fig. 3*C*). Compared with WT, TG (high) mice expressed markedly higher levels of the arginine and leucine transporters *Slc7a1*, *Slc7a3*, and *Slc7a5*; the glycine transporter *Slc6a9*; the cysteine transporter *Slc7a1*; and the d-serine transporter *Slc7a10*. In contrast to results in MEF studies (5, 20, 21), all genes involved in *de novo* serine biosynthesis, such as *Phgdh*, *Psat1*, and *Psph*, were highly induced. Serine is a precursor of other amino acids, such as glycine and cysteine. Glycine and cysteine are synthesized from serine by serine hydroxymethyltransferase (SHMT) and cystathionine-γ lyase (CTH), respectively. Along with glutamic acid, glycine and cysteine are precursors of GSH, the main antioxidant produced by cells. Glutamate-cysteine ligase, modified (GCLM), catalyzes the rate-limiting step in the synthesis of GSH from cysteine and glycine. Notably, all these enzymes (SHMT, CTH, and GCLM), that are essential for GSH synthesis, were induced in TG (high) mice. Furthermore, measuring the free amino acid levels in skeletal muscle cells showed no differences between serine levels in WT and TG (high) mice in contrast to the significant differences observed in glycine and arginine levels in TG (high) mice *vs.* those in WT mice (Fig. 3*D*). We then quantified GSH within the cells and found that GSH levels were higher in TG (high) mice than in WT mice (Fig. 3*E*). In TG mice, several, but not all, genes involved in *de novo* serine biosynthesis were induced. Therefore, the GSH levels were similar to those in WT mice, suggesting that the observed increase in GSH synthesis requires relatively strong eIF2α phosphorylation (Supplemental Fig. 1*E*, *F*). Taking all results together, we conclude that the increased serine as a result of increased synthesis and transport is not stored in intracellular amino acid pools but is converted to glycine and cysteine and utilized for GSH synthesis. It is well known that acute and prolonged exercise promotes the production of reactive oxygen species (ROS) (22). Expression of antioxidants such as GSH at the optimal time and concentration is critical for the adaptation of the muscle to exercise-induced oxidative stress (23, 24). We assumed that the eIF2α phosphorylation signal increases the level of GSH needed for adaptation to excise-induced muscle damage by profound up-regulation of serine biosynthesis.

### Phosphorylation of eIF2α in skeletal muscles induces the ATF4-mediated secretion of FGF21, an antiobesity myokine

**Figure 4.**
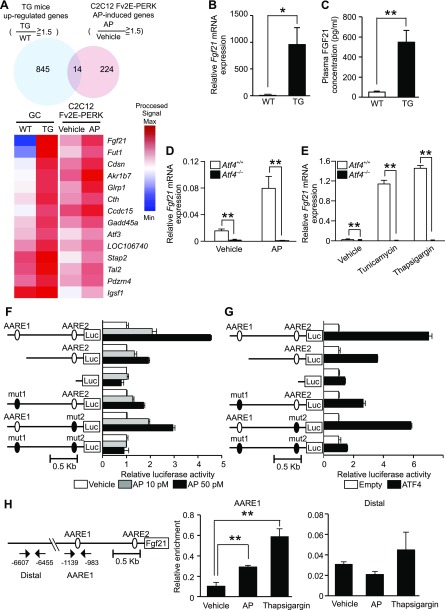
FGF21 induction by the PERK-eIF2α-ATF4 component of UPR*. A*) Top: Venn diagram (*n* = 2) showing the overlap between genes consistently expressed at a higher level in the TG(high) mice *vs.* WT mice or the AP-induced genes in C2C12 cells stably expressing Fv2E-PERK. Mice were euthanized at 8 wk. C2C12 cells expressing Fv2E-PERK were treated with 0.1 nM AP for 12 h. Bottom: heat map representation of the 14 genes found to be commonly expressed at an elevated level*. B*) RT-qPCR analysis (*n* = 6–8) of *Fgf21* mRNA expression in the gastrocnemius muscles of WT and TG mice (18-wk-old) fed a standard diet*. C*) Plasma FGF21 concentrations (*n* = 5) in WT and TG mice (18 wk old) fed a standard diet*. D*) RT-qPCR analysis (*n* = 3) of *Fgf21* mRNA expression in *Atf4*^+/+^ and *Atf4*^−/−^ MEFs stably expressing Fv2E-PERK at 8 h after treatment with 0.1 nM AP*. E*) RT-qPCR analysis (*n* = 3) of *Fgf21* mRNA expression in *Atf4*^+/+^ and *Atf4*^−/−^ MEFs at 8 h after stimulation with 2 μg/ml tunicamycin or 0.2 μM thapsigargin. *F*) Analysis of luciferase reporter activity (*n* = 3) of murine *Fgf21* promoters with deleted or mutated AAREs at 8 h after treatment with AP at the indicated doses in C2C12 cells expressing Fv2E-PERK. *G*) Analysis of luciferase reporter activity (*n* = 3) of murine *Fgf21* promoters with deleted or mutated AAREs in C2C12 cells expressing ATF4. *H*) Chromatin immunoprecipitation analysis (*n* = 3) of murine *Fgf21* promoters with the indicated primers and an anti-ATF4 antibody in C2C12 cells expressing Fv2E-PERK 5 h after treatment with 0.1 nM AP or 0.2 μM thapsigargin. Means ± sem. *P < 0.05, **P < 0.01 *vs.* WT mice.

Because we found more profound induction of amino acid metabolism regulation in skeletal muscles by eIF2α phosphorylation, we pursued the identification of novel downstream target genes of eIF2α phosphorylation signaling. However, the number of candidate genes from our mouse microarray analysis was relatively large (*n* = 859), and to narrow the group down further, we analyzed cell-autonomous gene expression in mouse myoblast C2C12 cells that varied in their levels of eIF2α phosphorylation and in the skeletal muscle of TG mice. C2C12 cells stably expressing Fv2E-PERK were treated with AP or vehicle. The results of microarray analysis identified 14 genes that were consistently expressed at a higher level in response to eIF2α phosphorylation (**Fig. 4*A***). The gene exhibiting the most prominent differential expression level was *Fgf21*, which is a known hepatokine that exerts antiobesity effects.

RT-qPCR analysis revealed that the level of *Fgf21* mRNA expression in TG skeletal muscles was ∼1000-fold higher than in WT mice (Fig. 4*B*). In accordance with its high mRNA expression, the plasma concentration of FGF21 was ∼10-fold higher in TG mice, as compared with that in WT mice (Fig. 4*C*). Next, we investigated the mechanism of *Fgf21* induction in C2C12 cells. When we treated the C2C12 cells stably expressing Fv2E-PERK with various concentrations of AP to induce different degrees of eIF2α phosphorylation, we observed a dose-dependent response, with increasing concentrations of AP leading to a proportional increase in FGF21 expression and secretion accompanied by an increase in eIF2α phosphorylation (Supplemental Fig. 1*A*–C). We showed that *Fgf21* mRNA expression was induced in C2C12 cells in response to the ER stress-inducing agents tunicamycin, thapsigargin, brefeldin A, and DTT (Supplemental Fig. 1*D*).

Given that the PERK-eIF2α pathway is known to induce gene expression *via* the ATF4 transcription factor (5), we used *Atf4*^−/−^ MEFs to investigate whether ATF4 is involved in the induction of *Fgf21* mRNA. ATF4 depletion almost completely abolished the transcriptional induction of *Fgf21* in both the AP-treated Fv2E-PERK system without ER stress and with ER stress caused by tunicamycin or thapsigargin, demonstrating that *Fgf21* induction was dependent on ATF4 (Fig. 4*D*, *E*).

To further analyze whether the transcriptional induction of *Fgf21* by ATF4 is caused by its direct interaction with the *Fgf21* promoter, we used *Fgf21* promoter reporters with deletions or mutations of amino acid response elements (AAREs), which are known to bind ATF4. AP-dependent eIF2α phosphorylation and ATF4 expression in transfected C2C12 cells increased the activation of the 1.5 kb murine *Fgf21* promoter reporter by ∼5- and 7-fold, respectively (Fig. 4*F*, *G*). When regions containing AARE1 and -2 were incrementally deleted, eIF2α phosphorylation- and ATF4-dependent reporter activation gradually became undetectable. Furthermore, promoter activation decreased when AARE1 or -2 were individually mutated and became undetectable when both sequences were mutated. To determine whether ATF4 directly binds to the *Fgf21* promoter and activates its transcription, we performed chromatin immunoprecipitation analysis in C2C12 cells. We confirmed the binding of ATF4 to the AARE1 in the *Fgf21* promoter in either an AP- or thapsigargin-dependent manner (Fig. 4*H*). These findings led us to conclude that eIF2α phosphorylation in skeletal muscles causes transcriptional induction to increase the secretion of FGF21 through direct promoter binding of ATF4.

### Phosphorylation of eIF2α in skeletal muscles suppresses fat accumulation by stimulating energy consumption in BAT, but not in skeletal muscles

**Figure 5.**
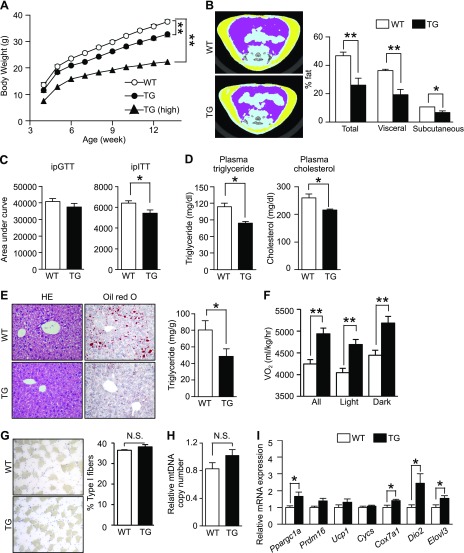
Effect of increased eIF2α phosphorylation in skeletal muscles on obesity*. A*) Body weight (*n* = 9–12); 2-way ANOVA) of WT, TG, and TG (high) mice fed an HFD from 4 wk of age*. B*) Typical CT images at the L5 vertebral level and CT-estimated body fat compositions (*n* = 5) of WT and TG mice fed an HFD for 12 wk. Area under the curve from results of intraperitoneal glucose (GTT) and insulin (ITT) tolerance tests (*n* = 4–5) (*C*) and plasma triglycerides and plasma cholesterol (*n* = 4–5) (*D*) in WT and TG mice fed an HFD for 12 wk*. E*) Typical H&E and Oil Red O staining and triglyceride contents (*n* = 4) per weight of the liver of WT and TG mice fed an HFD for 12 wk and levels of liver triglycerides. *F*) *V*o_2_ (*n* = 4) of WT and TG mice fed an HFD for 4 wk. *G*) Representative immunostaining of soleus muscle slow myosin heavy chain and percentage of type I muscle fibers (*n* = 4) of WT and TG mice fed an HFD for 12 wk. *H*) Analysis by qPCR of mitochondrial DNA (*n* = 5) in the tibialis anterior of WT and TG mice fed HFD for 12 wk. The data were corrected for contamination with genomic DNA. *I*) RT-qPCR (*n* = 5–6) analysis of mRNA expression of genes related to mitochondrial energy consumption in the BAT of WT and TG mice fed an HFD for 4 wk. Means ± sem. *P < 0.05, **P < 0.0 *vs*. WT mice.

FGF21 is a hormone secreted by various organs, including the liver and adipose tissue, and it regulates energy homeostasis through endocrine, autocrine, and paracrine actions (25, 26). For example, transgenic mice that overexpress *Fgf21* and mice administered FGF21 resist diet-induced obesity (27). In contrast, *Fgf21*-knockout mice become obese and glucose intolerant (28). Given these findings, we suspected that FGF21 secreted from the skeletal muscles of Fv2E-PERK TG mice would act as a myokine and stimulate energy consumption in BAT. To examine this hypothesis, we fed an HFD to WT, TG, and TG (high) mice. Although the food intake of TG mice was higher than that of WT mice (Supplemental Fig. *3D*), we observed that body weight gain was lower in TG mice than in WT mice (**Fig. 5*A***). The results of the CT revealed that TG and TG (high) mice exhibited lower visceral and subcutaneous fat accumulation than did WT mice (Fig. 5*B*; Supplemental Fig. 3*E*). To assess the impact of glucose metabolism status, we performed intraperitoneal glucose and insulin tolerance tests in WT, TG, and TG (high) mice. There was no significant difference in glucose tolerance between WT and TG mice; however, insulin resistance was lower in TG mice fed an HFD than in WT mice (Fig. 5*C*; Supplemental Fig. 3*A*, *B*). TG (high) mice displayed a greater degree of improvement in glucose tolerance and insulin resistance than TG mice (Supplemental Fig. 3*F*, *G*). Accordingly, plasma triglyceride and cholesterol levels in TG mice were lower than those in WT mice (Fig. 5*D*). Subsequently, we investigated fat accumulation in the liver, as it supplies lipids to adipose tissues. H&E staining, Oil Red O staining, and quantification of liver triglycerides revealed that the hepatic steatosis observed in WT mice induced by the HFD was markedly suppressed in TG mice (Fig. 5*E*).

TG mice fed an HFD were leaner than WT mice fed an HFD, despite their increased food intake. We hypothesized that increased energy expenditure is responsible for their leaner phenotype. As we expected, oxygen consumption (*V*o_2_) and energy consumption were higher in TG and TG (high) mice (Fig. 5*F*; Supplemental Fig. 3*C*, H) than in WT mice. To reveal the metabolic change in skeletal muscles, we investigated the proportion of type I fibers, which contain many mitochondria and play a central role in energy metabolism, and determined mitochondrial DNA copy numbers by PCR (Fig. 5*G*, *H*). There were no significant differences between the skeletal muscles of WT and TG mice. RT-qPCR analyses also revealed no significant differences in the expression levels of *Ppargc1a* and *Nrf1*, whose products regulate mitochondrial biogenesis, as well as those of *Pdk4*, *Cycs*, *Ndufs8*, and *Ucp3*, which encode proteins involved in mitochondrial energy metabolism, or those of *Cd36*, *Cpt1b*, and *Acadl*, whose products import and catalyze the β-oxidation of fatty acids (Supplemental Fig. 2*H*). To eliminate the possible metabolic regulation *via* an interorgan communication system, we also performed metabolic flux assays on C2C12 myoblast cells that stably expressed Fv2E-PERK and had been induced to differentiate. However, even when treated with AP to promote eIF2α phosphorylation, changes in mitochondrial respiration or fatty acid β-oxidation changes were not detected (Supplemental Fig. 2*I*, *J*). These results indicate that the skeletal muscle is not the tissue responsible for the increased energy expenditure.

Heat-producing BAT is one of the target tissues of FGF21 with respect to its antiobesity effect; therefore, we investigated energy metabolism in BAT. RT-qPCR analyses demonstrated that the expression levels of *Ppargc1a*, which regulates mitochondrial biogenesis, and those of *Cox7a1*, *Dio2*, and *Elovl3*, which influence heat production in BAT, in the TG mice were higher than those in WT mice (Fig. 5*I*). Moreover, a higher rectal temperature than in WT mice was observed only in TG (high) mice (Supplemental Fig. 3*I*). These findings suggest that phosphorylation of eIF2α in skeletal muscles suppresses fat accumulation by stimulating energy consumption through BAT mitochondrial activation.

### Cell-based screening identified a new small molecule, RDR03027, which induced eIF2α phosphorylation and expression of amino acid metabolism genes and *Fgf21*

**Figure 6.**
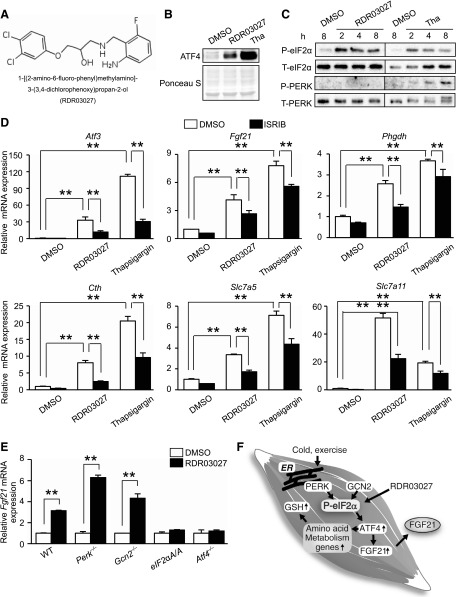
**A** novel small molecule activates eIF2α phosphorylation*. A*) Structure of hit compound RDR03027*. B*) Representative immunoblots of ATF4 and Ponceau staining of total protein in the nuclear fraction at 2 h after treatment with 40 μM RDR03027 or 0.2 μM thapsigargin in C2C12 cells*. C*) Representative immunoblots of phosphorylated and total eIF2α, phosphorylated and total PERK at the indicated times after treatment with 40 μM RDR03027 or 0.2 μM thapsigargin in C2C12 cells*. D*) RT-qPCR analysis (*n* = 3) of the expression of mRNA encoding targets of eIF2α phosphorylation at 8 h after treatment with 40 μM RDR03027 or 0.2 μM thapsigargin or/and 0.5 μM ISRIB in C2C12 cells*. E*) RT-qPCR analysis (*n* = 3) of *Fgf21* mRNA expression in WT, *Perk*^−/−^, *Gcn2*^−/−^, eIF2α^A/A^, and *Atf4*^−/−^ MEFs at 8 h after stimulation with 40 μM RDR03027. *F*) Schema of the study. Means ± sem. **P < 0.01 *vs*. WT.

The importance of eIF2α phosphorylation signaling prompted us to identify small molecules to regulate metabolism *via* FGF21. To identify the new inducer of eIF2α phosphorylation, we established an HEK293A cell line that stably expressed 3xAARE-luciferase. The sensitivity of this reporter in the cell line was confirmed by thapsigargin and histidinol. In this study, we screened 4000 chemical compounds and identified 120 molecules that altered eIF2α phosphorylation (Supplemental Fig. 4*A*). The hit compounds were subjected to second and third screenings with the 3xAARE-luciferase reporter and *Fgf21* promoter-luciferase reporter in C2C12 cells. In this study, we chose 1 hit compound, 1-[(2-amino-6-fluoro-benzyl)amino]-3-(3,4-dichlorophenoxy)propan-2-ol (RDR03027), for further analysis (**Fig. 6*A***; Supplemental Fig. 4*B*, *C*, *E*). To date, several compounds such as 3-(2,3-dihydrobenzo[*b*][1,4]dioxin-6-yl)-5,7-dihydroxy-4*H-*chromen-4-one, salubrinal, and guanabenz have been reported to up-regulate eIF2α phosphorylation signaling (29–31). However, RDR03027 does not share the basic structure to the 3 previously reported compounds. To validate the effect of RDR03027 on eIF2α phosphorylation signaling, induction of ATF4 and eIF2α phosphorylation were observed by immunoblot analysis. We confirmed the increase in ATF4 and eIF2α phosphorylation in C2C12 cells treated with RDR03027 (Fig. 6*B*, *C*). This compound did not induce the phosphorylation of PERK, suggesting that it did not trigger ER stress. To determine whether RDR03027 can activate the ISR, induction of ISR target genes was assessed by RT-qPCR. The up-regulation of *Atf3, Fgf21, Phgdh, Cth, Slc7a5,* and *Slc7a11* occurred in RDR03027-treated C2C12 cells. ISRIB was identified as an inhibitor of ISR signaling, which acts by blocking the translational attenuation mediated by eIF2α phosphorylation (32). We took advantage of ISRIB to test whether RDR03027-induced mRNA up-regulation is solely ISR dependent. ISRIB partially blocked RDR03027-induced up-regulation of the ISR target genes and ATF4 induction caused by RDR03027 in C2C12 cells (Fig. 6*D*; Supplemental Fig. 4*D*). However, ISRIB failed to completely block ATF4 induction caused by thapsigargin, and it remains unknown whether the RDR03027-induced *Fgf21* mRNA induction is mediated solely by eIF2α phosphorylation or by an alternative pathway. Therefore, we determined whether *Fgf21* mRNA induction caused by RDR03027 is dependent on eIF2α phosphorylation by using ISR-deficient MEFs. RT-qPCR revealed that *Fgf21* mRNA induction by RDR03027 was abolished in *eIF2α^A/A^* (homozygous mutant of the phosphorylation site) and *Atf4^−/−^* MEF cells, indicating that the effect of RDR03027 is mediated by eIF2α phosphorylation (Fig. 6*E*). RDR03027 induced *Fgf21* mRNA without PERK and GCN2, suggesting that an alternate eIF2α kinase or phosphatase could be regulated by RDR03027. These observations suggest that RDR03027 is a new ISR inducer that can induce *Fgf21* mRNA expression.

## DISCUSSION

UPR is involved, not only in proteostasis, but also in cell-autonomous noncanonical metabolic regulation (3, 4). In our study, when eIF2α was phosphorylated through PERK activation in skeletal muscles, it acted to promote energy consumption in BAT in a non–cell-autonomous manner *via* ATF4-mediated FGF21 secretion (Fig. 6*F*). Furthermore, eIF2α phosphorylation increased antioxidant GSH levels through increased expression of amino acid metabolism and transport genes. Other studies have shown that the PERK-eIF2α-ATF4 pathway induces GSH synthesis and increases the expression of a cysteine transporter, which increases antioxidant activity and facilitates adaptation to ER stress (5, 20, 21). In this study, we found that eIF2α phosphorylation in skeletal muscles increased a broader range of serine metabolism–related genes needed for GSH synthesis than previously observed in MEFs. These findings highlight the skeletal muscle–specific role of eIF2α phosphorylation in metabolic regulation.

Our results indicated that skeletal muscle–secreted FGF21 was responsible for resistance to HFD-induced obesity. Supporting our conclusion, the inhibited growth and impaired thrive phenotype of TG (high) mice is similar to that of *Fgf21* transgenic mice (27, 33). FGF21 secreted from white adipose tissue has been reported to act locally in an autocrine or paracrine instead of an endocrine manner (34). However, our data demonstrated that FGF21 secreted from TG mice did not work in skeletal muscles but in BAT as a myokine. FGF21 signaling requires the FGF receptor and β-Klotho as its receptor, and both are barely expressed in skeletal muscles (35), which could explain the lack of any obvious metabolic improvement in skeletal muscles of TG mice or in C2C12 cells. It is conceivable that fat accumulation in liver and white adipose tissue are ameliorated by increased FGF21 secretion and by BAT. Mice deficient in skeletal muscle autophagy promote whole-body energy metabolism and confer resistance to obesity through *Fgf21* expression (36), similar to that seen in our TG mice. Autophagy is largely known to contribute to amino acid homeostasis and to the quality control of mitochondria. Kim *et al.* (36) showed that the blocked clearance of damaged mitochondria in autophagy causes impaired activity of mitochondrial oxidative phosphorylation, which increases FGF21 expression through the induction of ATF4 as a mitokine. It has been reported that the eIF2α-ATF4 pathway is involved in the fine-tuning of the autophagy gene transcription program (37). Collectively, ER stress and autophagy-mediated mitochondria stress intersect at eIF2α phosphorylation. However, the mechanism that links ER stress and mitochondria stress to phosphorylation of eIF2α remains unclear. Autophagy and ISR signaling are essential for amino acid homeostasis, which may provide clues for further investigations into these associations.

Exercise-induced ER stress in skeletal muscles has been shown in our and previous studies. Intensive exercise is believed to increase ROS production as a result of the increased *V*o_2_ caused by muscle contraction (22). However, given that excessive or prolonged exposure to ROS could be detrimental, regulation of antioxidant enzymes such as superoxide dismutase, catalase, and glutathione peroxidase, as well as and nonenzymatic antioxidants such as GSH, is critical for the adaptation of the skeletal muscle to exercise (22). Indeed, a decrease in intracellular GSH/GSSG ratios was found in skeletal muscles after strenuous exercise (38, 39). We now show that eIF2α phosphorylation signaling increases GSH through increases serine biosynthesis, which could be a muscle adaptation to exercise-induced oxidative stress. Our study showed that endurance capacity was similar between WT and TG animals, despite a reduction in muscle strength of TG mice, suggesting that endurance capacity might be improved in TG mice. To clarify this point, a more appropriate *in vivo* model is needed to separate muscle endurance capacity and muscle mass. In addition to endurance capacity, constitutive GSH production induced by eIF2α phosphorylation may alleviate insulin resistance in skeletal muscles. Another report showed that aging rodents and humans have a deficiency in GSH and insulin resistance in skeletal muscles, and GSH restoration by dietary glycine and cysteine supplementation leads to a recovery from insulin resistance (40). Although increased thermogenesis was not observed in skeletal muscles of TG mice, the increased GSH may have attenuated insulin resistance in skeletal muscles of TG mice during HFD feeding in addition to the non–cell-autonomous *Fgf21* effects in TG mice.

Overexpression of FGF21 sensitized mice to torpor and hibernation-like metabolic changes. Indeed, several studies have shown that FGF21 regulates the thermogenic machinery that combats hypothermia (41, 42). FGF21 and ATF4 are both increased in hibernating animals that are fasting and exposed to cold temperatures (43, 44). Our study showed that cold exposure of mice resulted in an induction of the ATF4 downstream target genes and *Fgf21* in skeletal muscles (Fig. 1; Supplemental Fig. 2*E*, *F*). A single session of acute exercise resulted in no changes in FGF21 mRNA, suggesting that a single, short-term eIF2α-phosphorylating activity, such as exercise, is not enough for FGF21 induction in muscle and that sustained eIF2α phosphorylation such as cold exposure requires for FGF21 induction (Supplemental Fig. 2*G*). Indeed, an increase in eIF2α phosphorylation, which is upstream of ATF4 and FGF21, has been reported to occur during hibernation (45), and suppression of protein synthesis *via* eIF2α phosphorylation is suggested to be an important component of the hibernating state. Hypothermia and starvation are comparable to the stresses that occur in hibernating animals, and eIF2α phosphorylation–mediated thermogenesis *via* suppression of FGF21 and protein synthesis could be beneficial under these conditions. Taken together, these findings suggest that the eIF2α phosphorylation–FGF21 signaling axis contributes to the adaptive response during hypothermia and starvation. Further investigation is needed to verify these associations.

The skeletal muscle has a special type of smooth ER named the sarcoplasmic reticulum, which plays a critical role in muscle mechanics and metabolic homeostasis. Total gene knockout experiments have shown that ATF6α directly interacts with peroxisome proliferator-activated receptor-γ coactivator (PGC)1 and hence contributes a considerable part of PGC1 function in exercise tolerance in skeletal muscles (10). Together with our findings, this result suggests that the 3 branches of the UPR regulate skeletal muscle homeostasis in a parallel way, whereby overall UPR signaling provides an additional layer of regulation in skeletal muscles. The acquisition of more detailed knowledge of how the ISR signaling pathway regulates biologic functions will facilitate the development of drugs to intervene in pathologies caused by its dysfunction. We found that the newly identified small molecule RDR03027 increased eIF2α phosphorylation and induced the expression of amino acid metabolism genes, as well as *FGF21*. Unfortunately, *in vivo* experiments with RDR03027 were problematic because of limited bioavailability and must therefore await additional studies. ISR signaling can be activated by facilitating eIF2α kinase activity or by inhibiting the eIF2α phosphatase. Among the ISR activators, it has been reported that 3-(2,3-dihydrobenzo[*b*][1,4]dioxin-6-yl)-5,7-dihydroxy-4*H-*chromen-4-one activates PKR and PERK (29) and that both salubrinal and guanabenz inhibit the activity of eIF2α phosphatase (30, 31). The detailed molecular mechanism of eIF2α phosphorylation by RDR03027 remains to be determined; it may be facilitated by the development of a more soluble compound.

Using genetic manipulation to activate eIF2α phosphorylation in a single tissue (*i.e.,* skeletal muscle) without causing ER stress uncovered a novel aspect of tissue-specific eIF2α phosphorylation signaling. Defining the significance of the eIF2α phosphorylation signal in various organs is essential for establishing therapeutic effects and anticipating adverse side effects of drugs that target the signal. Accomplishing this goal may provide new avenues for therapeutic development.

## Supplementary Material

Supplemental Data

## Abbreviations

4EBP: eIF4E-binding protein
AARE: amino acid response element
ACC: acetyl-CoA carboxylase
AP: AP20187
ATF: activating transcription factor
BAT: brown adipose tissue
CT: computed tomography
CTH: cystathionine-γ lyase
*Edem*: ER degradation enhancer, mannosidase α-like
eIF: eukaryotic translation initiation factor
ER: endoplasmic reticulum
FGF: fibroblast growth factor
GCLM: glutamate-cysteine ligase, modifier
GCN: general control nonderepressible
*Grp*: glucose-regulated protein
GSH: glutathione
GSSG: oxidized glutathione
H&E: hematoxylin and eosin
HEK: human embryonic kidney
HFD: high-fat diet
IRE: inositol-requiring enzyme
ISR: integrated stress response
ISRIB: integrated stress response inhibitor
MEF: mouse embryonic fibroblast
OCR: oxygen consumption rate
PERK: protein kinase R-like ER kinase
PGC: peroxisome proliferator-activated receptor γ coactivator
PKR: protein kinase R
*Ppp1r15a*: protein phosphatase 1 regulatory subunit 15A
qPCR: quantitative PCR
ROS: reactive oxygen species
SHMT: serine hydroxymethyltransferase
*Slc*: solute carrier family
TG: transgenic
UPR: unfolded protein response
*V*o_2_: oxygen consumption
WT: wild-type
XBP: X-box binding protein
