# Supplementary material for: Skeletal muscle–specific eukaryotic translation initiation factor 2α phosphorylation controls amino acid metabolism and fibroblast growth factor 21–mediated non–cell-autonomous energy metabolism
Source: FASEB J. 2015 Oct 20;30(2):798–812. doi: 10.1096/fj.15-275990 (PMC4945323; doi:10.1096/fj.15-275990)
Supplement: Supplemental Data [file supp_30_2_798__index.html]

Skeletal muscle–specific eukaryotic translation initiation factor 2α phosphorylation controls amino acid metabolism and fibroblast growth factor 21–mediated non–cell-autonomous energy metabolism — Skeletal muscle–specific eukaryotic translation initiation factor 2α phosphorylation controls amino acid metabolism and fibroblast growth factor 21–mediated non–cell-autonomous energy metabolism — Supplemental Data 

# Skeletal muscle–specific eukaryotic translation initiation factor 2α phosphorylation controls amino acid metabolism and fibroblast growth factor 21–mediated non–cell-autonomous energy metabolism

## Supplemental Data

- Supplemental Data
