# Supplementary material for: Skeletal muscle–specific eukaryotic translation initiation factor 2α phosphorylation controls amino acid metabolism and fibroblast growth factor 21–mediated non–cell-autonomous energy metabolism
Source: FASEB J. 2015 Oct 20;30(2):798–812. doi: 10.1096/fj.15-275990 (PMC4945323; doi:10.1096/fj.15-275990)
Supplement: Supplemental Data [file supp_fj.15-275990_Supplemental_Figures.pdf]

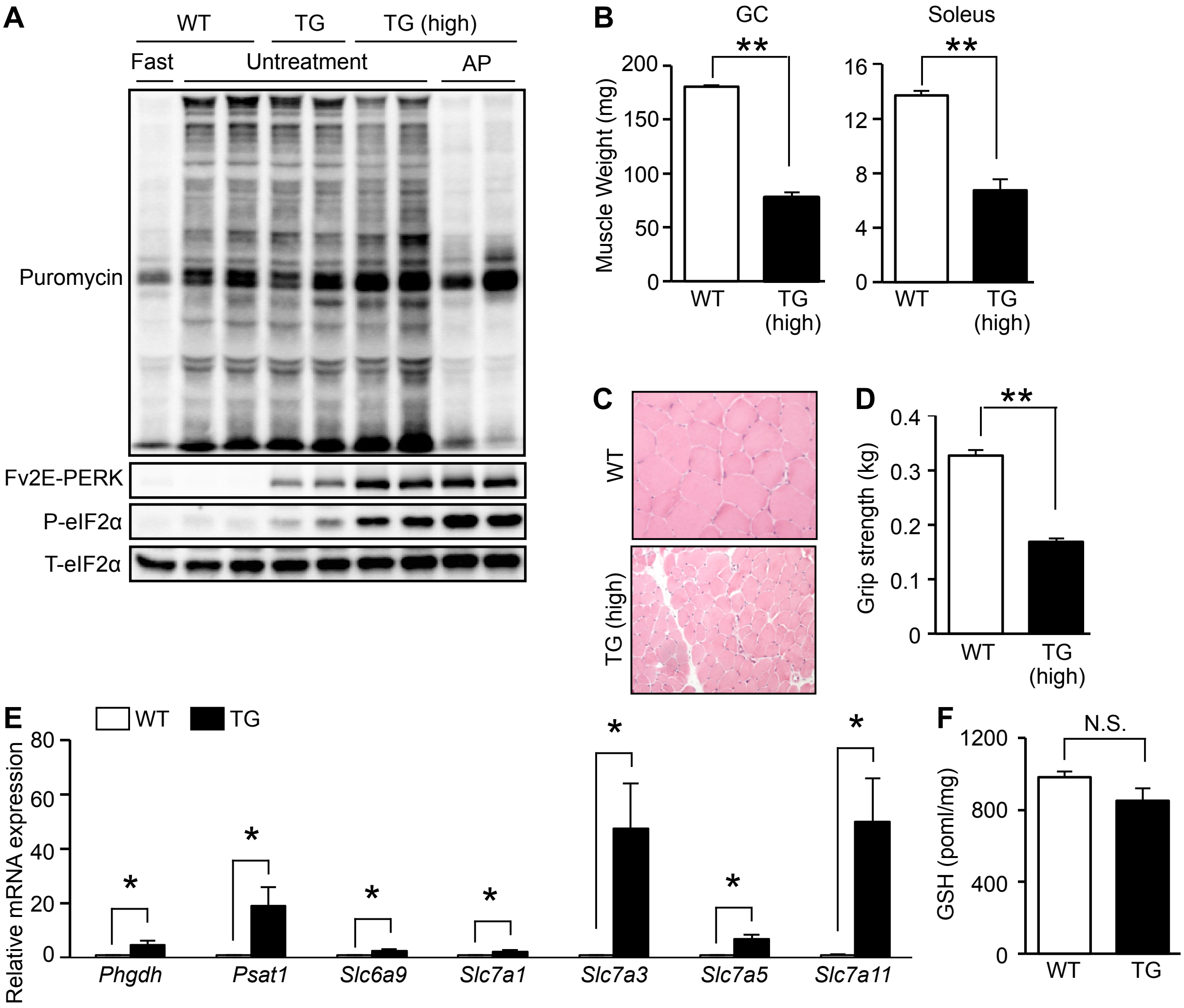

Supplemental Figure 1. Effects of constitutive high phosphorylation of eIF2α in mouse skeletal muscles.
(A) Immunoblots of puromycin-labeled peptide, phosphorylated eIF2α, total eIF2α, and Fv2E-PERK in gastrocnemius muscles of WT, TG and TG (high) mice. A part of WT mice were fasted for 48 h and a part of TG (high) mice were pre-treated with 100 ng/kg of AP for 1 h. All mice were intraperitoneally injected with 0.04 μmol/g of puromycin before 30 min of tissue collection.
(B) Muscle weight (mean ± SEM, n = 3, $^{**}p < 0.01$) of the gastrocnemius and soleus muscles of 10 month-old WT and TG (high) mice fed the standard diet.
(C) Representative HE staining of the tibialis anterior of WT and TG (high) mice fed the standard diet.
(D) Grip strength (mean ± SEM, n = 7-8, $^{*}p < 0.05$) of WT and TG (high) mice fed the standard diet.
(E) RT-qPCR analysis (mean ± SEM, n = 7, $^{*}p < 0.05$) of the expression of mRNAs encoding proteins involved in amino acid metabolism in gastrocnemius muscles of WT and TG mice fed the standard diet.
(F) Total GSH levels (means ± SEM, n = 4) in the gastrocnemius muscles of WT and TG mice fed the standard diet.

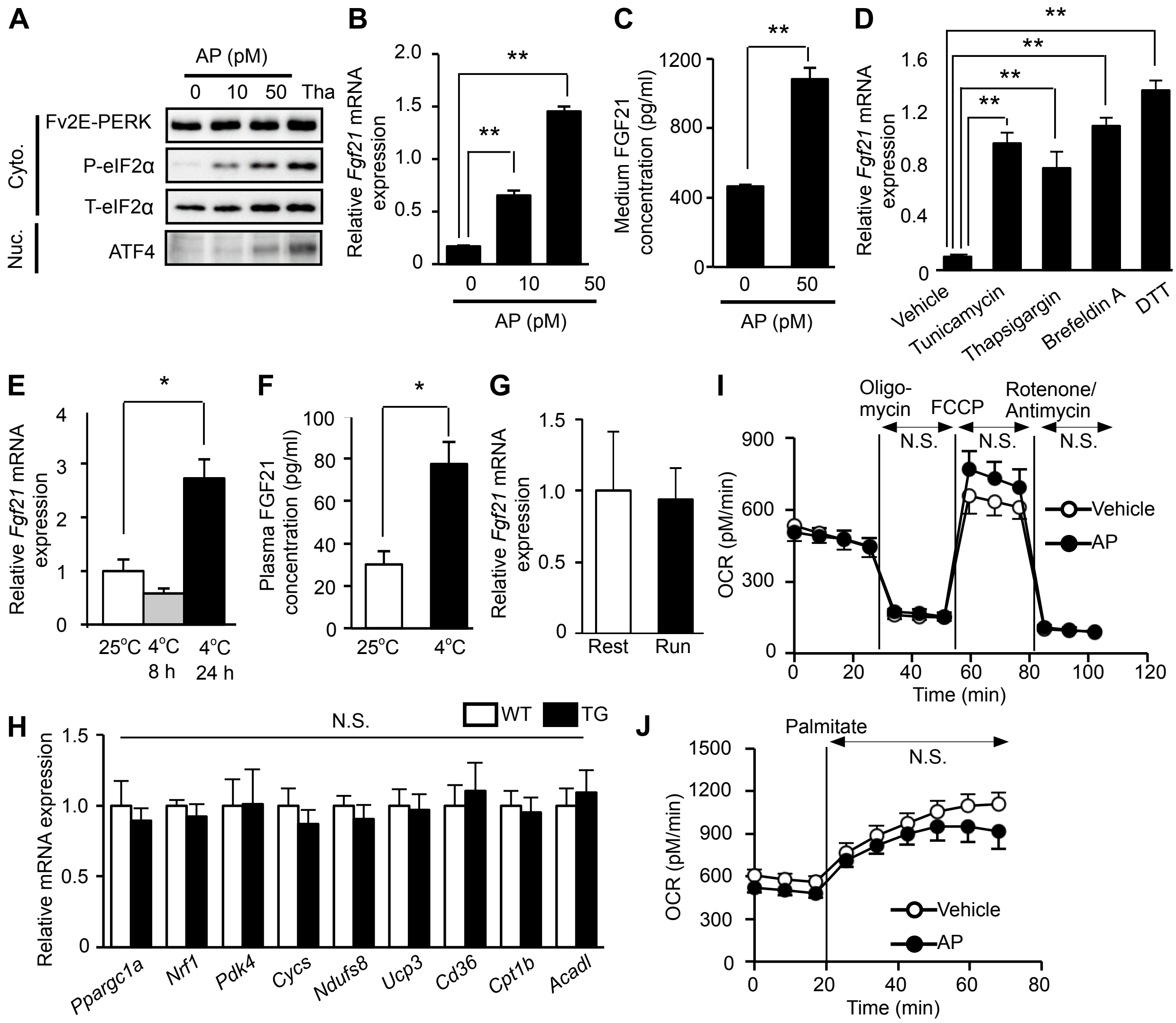

Supplemental Figure 2. Effects of constitutive high phosphorylation of eIF2α on FGF21 induction and mitochondrial energy consumption in mouse skeletal muscles.

(A) Immunoblots of Fv2E-PERK, phosphorylated eIF2α, and total eIF2α in the cytosolic fraction (Cyto.) and ATF4 in the nuclear fraction (Nuc.) 2 h after treatment with AP at the indicated dose or 0.2 μM thapsigargin in C2C12 cells stably expressing Fv2E-PERK.

(B) RT-qPCR analysis (mean ± SEM, n = 3, **p < 0.01) of Fgf21 mRNA expression at 8 h after treatment with AP at the indicated dose in C2C12 cells stably expressing Fv2E-PERK.

(C) The ELISA determined FGF21 concentration (mean ± SEM, n = 3, **p < 0.01) in culture medium 24 h after treatment with AP at the indicated dose in C2C12 cells stably expressing Fv2E-PERK.

(D) RT-qPCR analysis (mean ± SEM, n = 3, **p < 0.01) of Fgf21 mRNA expression at 8 h after treatment with various ER stress-inducing agents in C2C12 cells.

(E) RT-qPCR analysis (mean ± SEM, n = 5, *p < 0.05) of the Fgf21 mRNA expression in the gastrocnemius muscles of WT mice exposed to 4°C for the indicated time.

(F) Plasma FGF21 concentrations (mean ± SEM, n = 5, *p < 0.05) of WT mice exposed to 4°C for 24 h.

(G) RT-qPCR analysis (mean ± SEM, n = 5) of the Fgf21 mRNA expression in the lower limb skeletal muscles of mice during rest and at 5 h after one session of running on a treadmill.

(H) RT-qPCR analysis (mean ± SEM, n = 5, N.S. = not significant) of the transcription of genes related to mitochondrial energy consumption in the gastrocnemius muscles of WT and TG mice fed HFD for 12 weeks.

(I) OCR as a measure of fatty acid β-oxidation (means ± SEM, n = 3, N.S. = not significant) of vehicle- and 0.1 nM AP-treated (24 h) C2C12 cells stably expressing Fv2E-PERK after the sequential injection of 1 μM oligomycin (ATP synthase inhibitor), 1 μM carbonyl cyanide p-[trifluoromethoxy]-phenyl-hydrazone (uncoupling agent), 1 μM rotenone (complex I inhibitor), and 1 μM antimycin A (complex III inhibitor).

(J) OCR, after the injection of 200 μM palmitate, as a measure of fatty acid β-oxidation (mean ± SEM, n = 3, N.S. = not significant) of vehicle-treated and AP-treated C2C12 cells stably expressing Fv2EPERK.

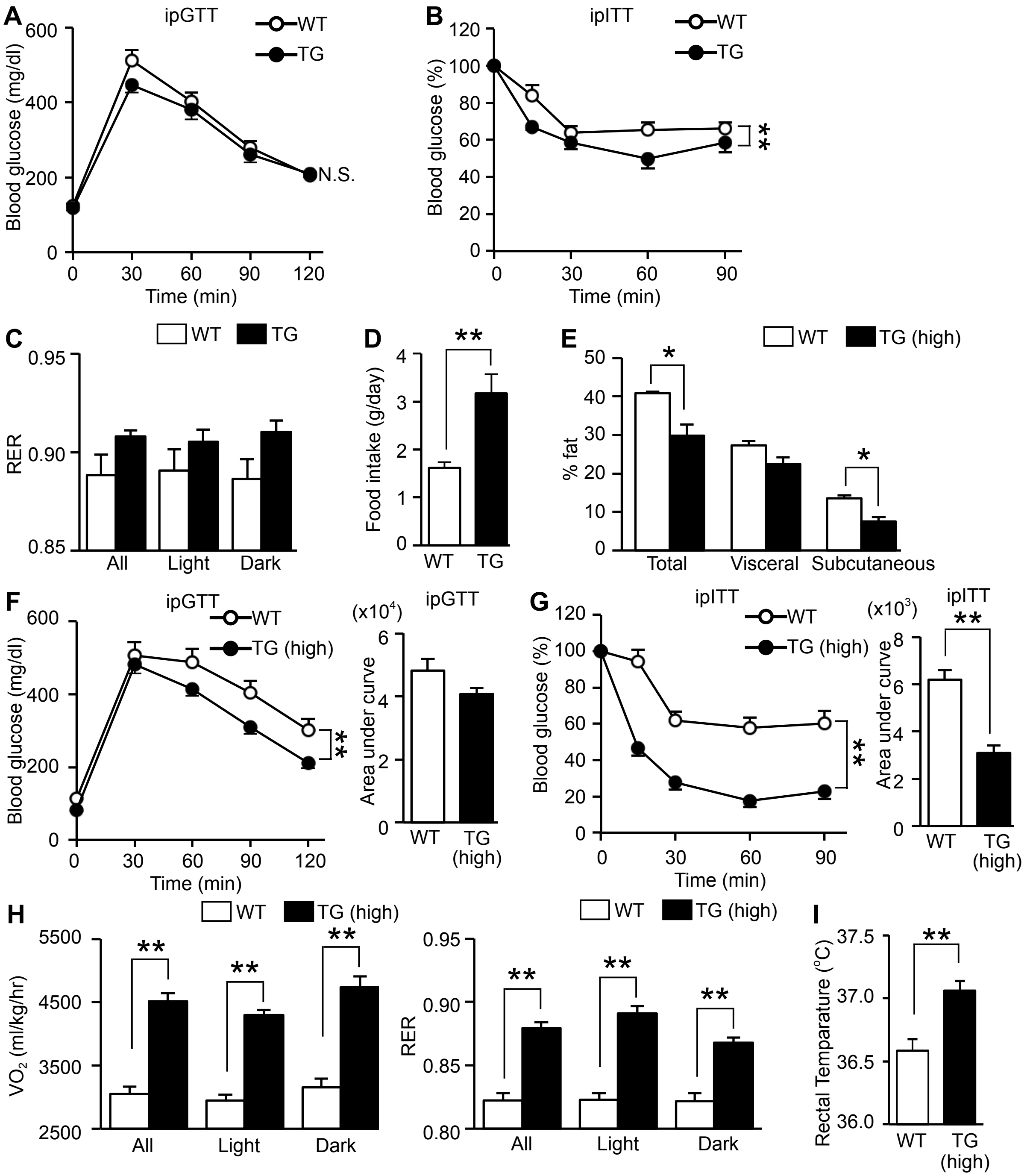

Supplemental Figure 3. Effect of increased eIF2α phosphorylation in skeletal muscles on obesity

(A) Result of intraperitoneal glucose tolerance test (mean ± SEM, n = 4–5, N.S. = not significant) in WT and TG mice fed HFD for 12 weeks.

(B) Result of intraperitoneal insulin tolerance test (mean ± SEM, n = 4–5, $^{**}p < 0.01$) in WT and TG mice fed HFD for 12 weeks.

(C) RER (mean ± SEM, n = 4) of WT and TG mice fed HFD for 4 weeks.

(D) Food intake of WT and TG mice (14 weeks old, n = 4, $^{**}p < 0.01$) fed a HFD.

(E) CT-estimated body fat compositions (mean ± SEM, n = 5, $^{*}p < 0.05$) of WT and TG(high) mice fed HFD for 12 weeks.

(F) Result and area under curve of i.p. glucose tolerance test (mean ± SEM, n = 4–5, $^{**}p < 0.01$) in WT and TG(high) mice fed HFD for 12 weeks.

(G) Result and area under curve of i.p. insulin tolerance test (mean ± SEM, n = 4–5, $^{**}p < 0.01$) in WT and TG(high) mice fed HFD for 12 weeks.

(H) VO2 and RER (mean ± SEM, n = 4, $^{**}p < 0.01$) of WT and TG(high) mice fed HFD for 4 weeks.

(I) Rectal temperature (mean ± SEM, n = 4, $^{**}p < 0.01$) of WT and TG(high) mice fed HFD for 4 weeks.

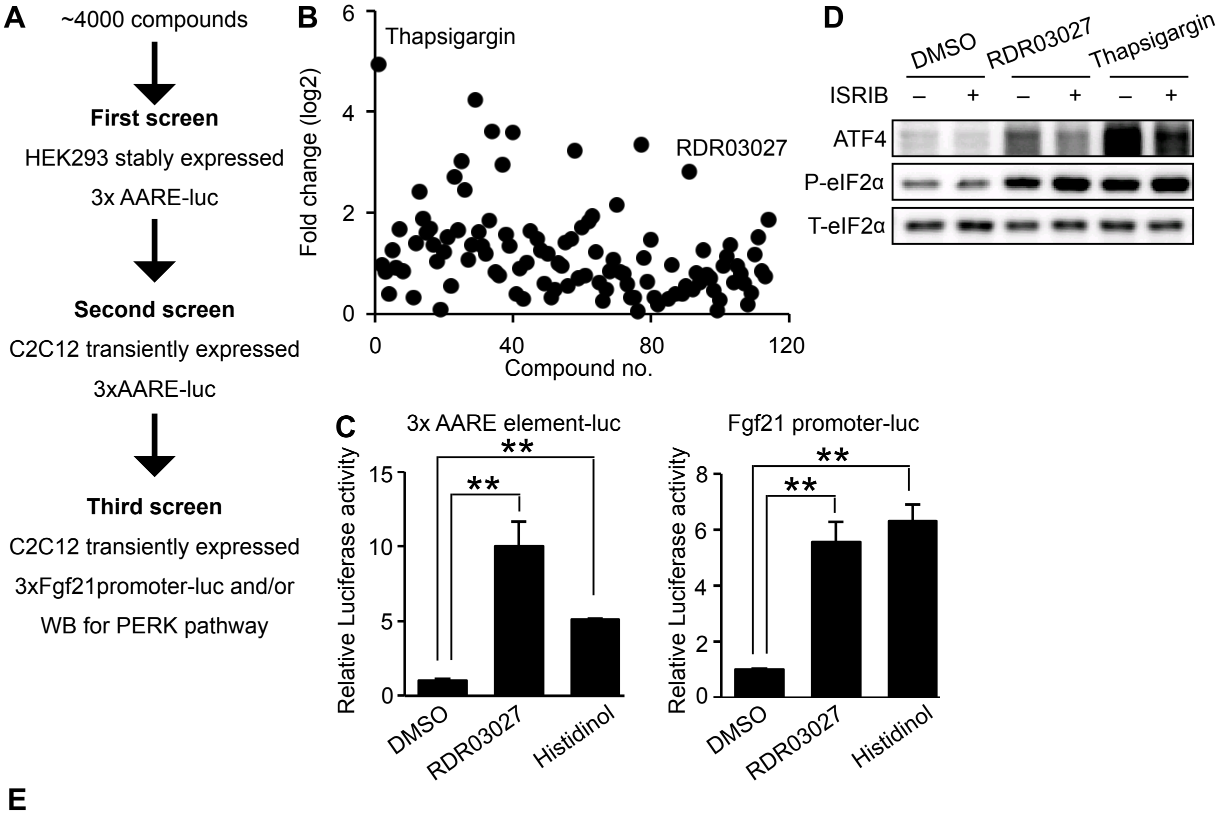

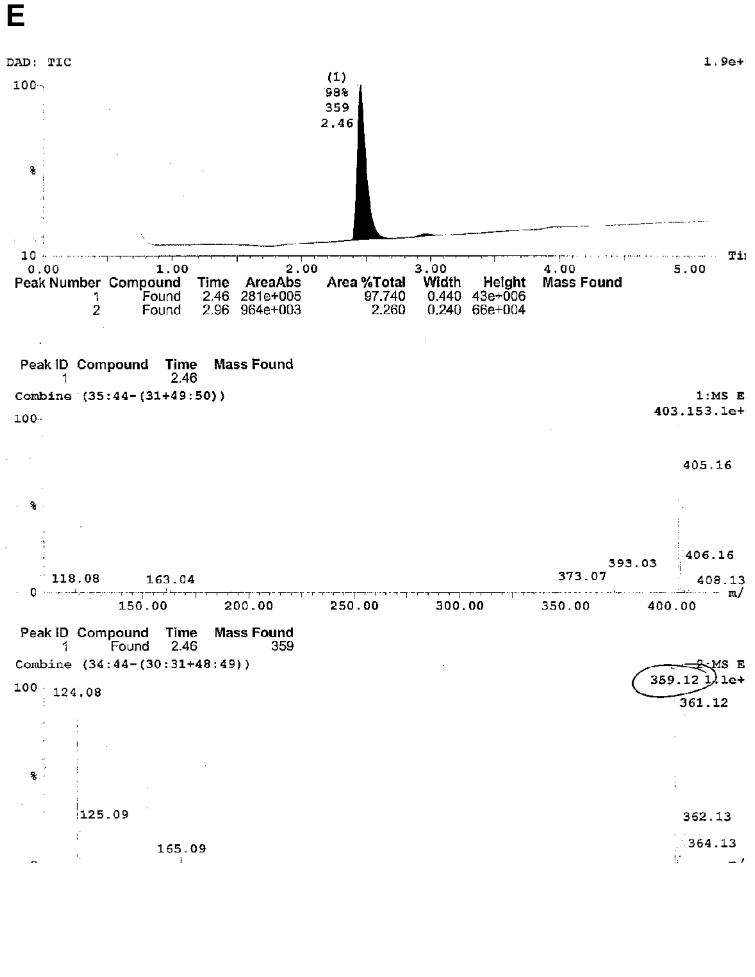

Supplemental Figure 4. Identification of a small molecule that induces Fgf21 mRNA via eIF2α phosphorylation.

(A) Schematic of screening to identification of a small molecule that induces eIF2α phosphorylation.

(B) Second screen results. C2C12 cells were transfected with 3xAARE luciferase reporter and treated with a library compound for 12 h. Thapsigargin treatment was used as the positive control.

(C) Analysis of luciferase reporter activity (mean ± SEM, n = 3, **$p < 0.01$) of 3xAARE and murine Fgf21 promoters in C2C12 cells treated with RDR03027. Histidinol was used as the positive control.

(D) Representative immunoblots of ATF4, phosphorylated and total eIF2α at 4 h after treatment with 40 uM RDR03027 and/or 0.5 μM ISRIB in C2C12 cells. Thapsigargin was used as the positive control.

(E) Analytical data for RDR03027. RDR03027 was purchased from Maybridge Chemical. The HPLC-DAD trace of RDR has the intense peak at retention time 2.46 min, suggesting 97.74% purity was achieved. The LC-MS spectrum shows a protonated molecular ion signal at m/z 359.12 in the positive mode, and the experimental molecular weight was compatible with the molecular formula ($C_{16}H_{17}Cl_2FN_2O_2$, MW 359.23).
